# Protoporphyrin Treatment Modulates Susceptibility to Experimental Autoimmune Encephalomyelitis in miR-155-Deficient Mice

**DOI:** 10.1371/journal.pone.0145237

**Published:** 2015-12-15

**Authors:** Jinyu Zhang, Michel Y. Braun

**Affiliations:** 1 National Engineering Research Center of Immunological Products, Department of Microbiology and Biochemical Pharmacy, College of Pharmacy, Third Military Medical University, Chongqing, PR China; 2 Institute for Medical Immunology, Faculty of Medicine, UniversitéLibre de Bruxelles (ULB), Gosselies, Belgium; Washington University, UNITED STATES

## Abstract

We previously identified heme oxygenase 1 (HO-1) as a specific target of miR-155, and inhibition of HO-1 activity restored the capacity of *miR-155* ^*-/-*^ CD4^+^ T cells to promote antigen-driven inflammation after adoptive transfer in antigen-expressing recipients. Protoporphyrins are molecules recognized for their modulatory effect on HO-1 expression and function. In the present study, we investigated the effect of protoporphyrin treatment on the development of autoimmunity in *miR-155*-deficient mice. MiR-155-mediated control of HO-1 expression in promoting T cell-driven chronic autoimmunity was confirmed since HO-1 inhibition restored susceptibility to experimental autoimmune encephalomyelitis (EAE) in *miR-155*-deficient mice. The increased severity of the disease was accompanied by an enhanced T cell infiltration into the brain. Taken together, these results underline the importance of miR-155-mediated control of HO-1 expression in regulating the function of chronically-stimulated T cells in EAE.

## Introduction

MicroRNAs (miRNA) are small non-encoded RNAs that have a potent impact on the homeostasis and function inimmune system [1–2]. MiR-155, the first miRNA to be studied in miRNA-deficient mice, was found to have pleiotropic effects on normal immune function. *MiR-155*-deficient mice are immunodeficient, displaying a severely disturbed germinal center (GC) response and dysregulated transcription factor, cytokine, and chemokine expression [3–4]. Overexpression of miR-155 in CD4^+^ T cells promotes Th1 differentiation by targeting IFN-γ recptor α chain (IFN-γRα) [5]. Importantly, a T cell-intrinsic expression of miR-155 was required for IL-17A production and inflammatory T cell development [6]. Moreover, miR-155 supports Treg homeostasis through the suppression of Suppressor of Cytokine Signaling 1 (SOCS1) [7].

Previously we have identified heme oxygenase 1 (*Hmox1*, HO-1) as a specific target of miR-155, and inhibition of HO-1 activity restored the capacity of *miR-155* ^*-/-*^CD4^+^ T cells to promote inflammation [8]. HO-1 is a rate-limiting intracellular enzyme that degrades heme into biliverdin and free iron and CO [9]. The reaction products have potent anti-inflammatory and anti-oxidative effect. Our previous study also showed that restoring function in chronically-stimulated CD4^+^ T cells would depend on the capacity of miR-155 to regulate HO-1 expression. HO-1 regulatory activity is centered on T cell proliferation and migration to sites of inflammation. We then questioned whether this observation could be extended to other systems where miR-155 was shown to control the activity of chronically-stimulated T cells. The regulatory role played by miR-155 on T cell-mediated chronic inflammation is well established in mouse experimental autoimmune encephalomyelitis (EAE) model [6, 10]. Myelin oligodendrocyte glycoprotein peptide fragment 35–55 (MOG_35-55_) immunization upregulated the expression of miR-155 in T cells, and *miR-155*-deficient mice were highly resistant to EAE [6]. In addition, induction of HO-1 by metal protoporphyrins or exposure to its product CO would arrest EAE progression and *Hmox1*-deficient mice are more sensitive to EAE. The failure to attenuateEAE progression in *Hmox1* ^*-/-*^ mice by HO-1 activation confirmed that the protective effect requires the expression of HO-1 [11–12]. Thus, in the present study, we intended to investigate whether the resistance of *miR-155* ^*-/-*^ mice to EAE could be lifted by the inhibition of HO-1. Treatment with the HO-1 inhibitor Zn (II) Protoporphyrin IX (ZnPP) restored susceptibility to EAE in *miR-155*-deficient mice. Increased severity of the disease was accompanied by an enhanced T cell infiltration of the brain. Taken together, these results underline the importance of miR-155-mediated control of HO-1 expression in regulating the function of autoimmune T cells in EAE.

## Materials and Methods

### Mice

*Rag1* ^*-/-*^ and *miR-155* ^*+/+*^B6 congenic mice were purchased from Charles River. *MiR-155* ^*-/-*^ B6 mice were purchased from Jackson Laboratories. All age and sex matched mice used in this study were between 6–10 weeks old and were bred in individually ventilated cages on the same rack under specific pathogen-free conditions (FELASA) at the Institute for Medical Immunology, UniversitéLibre de Bruxelles, Belgium.

### EAE

EAE was induced in mice aged from 6–8 weeks old. The detailed protocols for immunization and scoring were described previously [13]. In adoptive transfer experiments, spleen CD4^+^ T cells from *miR-155* ^*-/-*^ mice were collected by positive selection with FITC-conjugated anti-mouse CD4 (BD Biosciences), followed by anti-FITC magnetic beads (Miltenyi Biotec) and sorted by magnetic sorting according to manufacturer’s protocol [13]. Purified *miR-155* ^*-/-*^CD4^+^T cells were injected i.v (4x10^6^ cells/mouse) into *Rag1* ^*-/-*^ recipients. Recipient mice were subjected to EAE induction 24 hours after the transfer. In all experiments, mice were sacrificed at the peak of the diseases for analysis.

### Leukocyte isolation from brain

Leukocytes contained in brain infiltrates were isolated as previously described [13–14]. In brief, brains were digested by collagenase D (2.5 mg/ml, Roche) and DNaseI(100 μg/ml, Roche) for 30min at 37°C with rotation, followed by 30/70 percoll (GE Healthcare Life Sciences) gradient centrifugation.

### Protoporphyrin treatment

Zn (II) Protoporphyrin IX (ZnPP) and Co (III) protoporphyrin IX chloride (CoPP) (Frontier Scientific Porphyrin Products) were dissolved as previously described [8]. Solution without protoporphyrin was used as vehicle. ZnPP or CoPP was administered i.p every 2 days (200 μl/mouse, 5 mg/kg). For the EAE experiment, the protoporphyrin injection was started at the immunization with MOG_35-55_. For *miR-155* ^*-/-*^ CD4^+^ T cell adoptive transfer experiments, ZnPP injection was started at the time of the transfer.

### Flow cytometry

Single cell suspensions of spleen, draining lymph node (inguinal region) and brain from EAE mice were collected at the indicated time, the detailed antibodies and methods for surface and intracellular staining and analysis were described previously [13].

### Statistical analysis

Results were presented as Mean ±SEM. Mann-Whitney’s U test was used to compare two groups. For multiple group comparisons, one-way ANOVA tests were performed followed up by Tukey’s post-hoc multiple comparisons test. P< 0.05 was considered statistically significant.

### Ethics statement

This study was carried out in strict accordance with the Royal Decree (N°2013024221) of the 29th of May 2013 on the protection of experimental animals and published on the 10th of July 2013 in the Belgium code of law. The protocol was approved by the Comité d’Ethique et du Bien-Etre Animal of the Institute for Medical Immunology of the Université Libre de Bruxelles (Permit Number: LA1500518). All surgery was performed under xylazine/ketamine anesthesia, and all efforts were made to minimize suffering.

## Results

### The resistance of *miR-155* ^*-/-*^ mice to EAE was lifted by HO-1 inhibition

**Fig 1:**
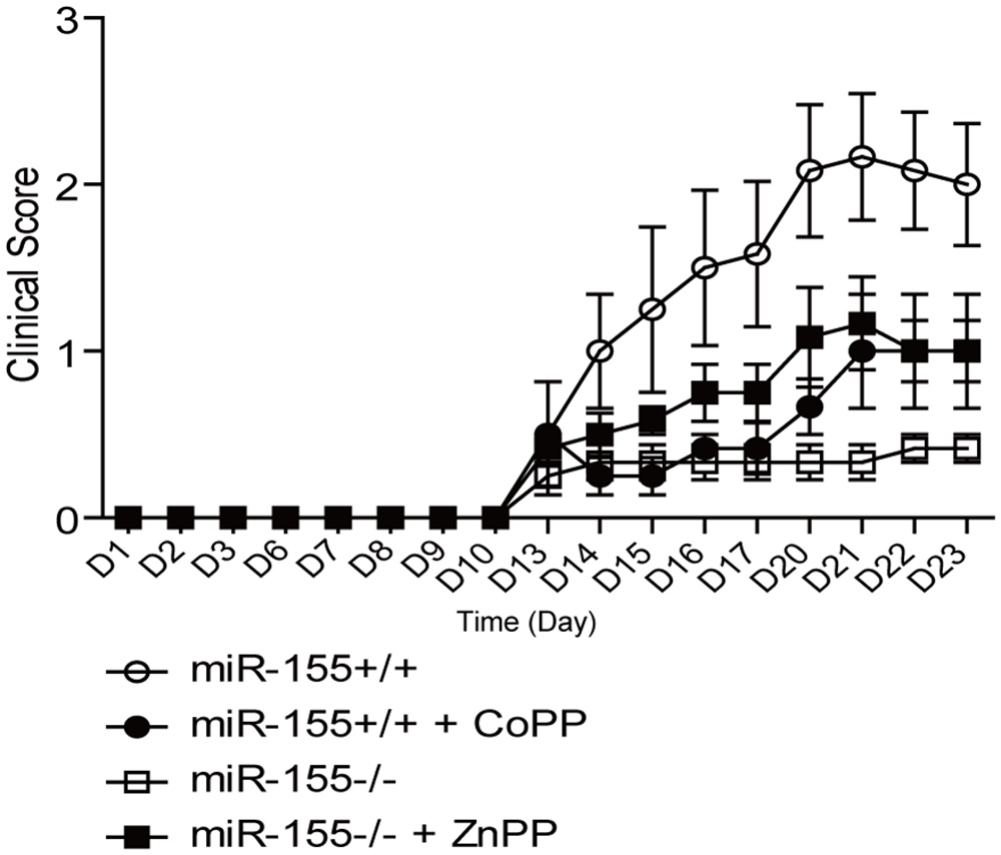
The resistance of *miR-155* ^*-/-*^ mice to EAE was lifted by HO-1 inhibition. EAE was induced in *miR-155* ^*+/+*^ and *miR-155* ^*-/-*^ mice that received CoPP, ZnPP or vehicle alone. Disease severity was scored based on clinical symptoms (n = 7–10). Data are shown as Mean ±SEM. Data are representative of two independent experiments. Statistical analysis was done with the Mann-Whitney U-test.

We have previously identified heme oxygenase 1 (HO-1) as a specific target of miR-155, and inhibition of HO-1 activity restored the capacity of *miR-155* ^*-/-*^CD4^+^ T cells to promote inflammation [8]. We then tested whether this observation could be extended to other systems where miR-155 was shown to control the activity of chronically-stimulated T cells. The regulatory role played by miR-155 on T cell-mediated chronic inflammation is well established in mouse experimental autoimmune encephalomyelitis (EAE) model [6, 10]. Thus, experimental protocols were designed to identify a possible role for miR-155 in HO-1-mediated regulation of T cell activity in EAE. Both *miR-15*5^*+/+*^ and *miR-155* ^*-/-*^mice were immunized with MOG_35-55_ peptide. As anticipated, *miR-155* ^*-/-*^mice displayed a lower peak of disease severity, in contrast, *miR-155* ^*+/+*^mice exhibited a more pronounced pathology (mean maximal score 2.17±0.38 vs 0.33±0.11, p<0.05 by two tailed Mann-Whitney U test). In order to determine whether HO-1 was responsible for the incapacity of *miR-155*-deficient mice to develop EAE, we injected repeatedly HO-1 inhibitor ZnPP in MOG_35-55_ peptide-immunized *miR-155* ^*-/-*^ mice and compared with animals injected with vehicle for the development of EAE. As shown in Fig 1, HO-1 inhibition worsened significantly the clinical signs of EAE in *miR-155* ^*-/-*^ mice (mean maximal score 1.16±0.27 vs 0.33±0.11, p<0.05 by two tailed Mann-Whitney U test). This observation was consistent with a role for miR-155 in repressing HO-1-mediated regulatory process in the development of EAE. In agreement with what has been published previously [11], induction of HO-1 expression and function by CoPP administration reversed paralysis (mean maximal score 2.17±0.38 vs 1.00±0.34, p<0.05 by two tailed Mann-Whitney U test). (Fig 1). On the contrary, injecting HO-1 inhibitor to *miR-155* ^*+/+*^ mice did not enhance disease pathology (S1 Fig). Thus, worsened disease caused by HO-1 inhibition was only observed in *miR-155* ^*-/-*^ mice.

### Inhibition of HO-1 promote T cell infiltrated into the brain

**Fig 2:**
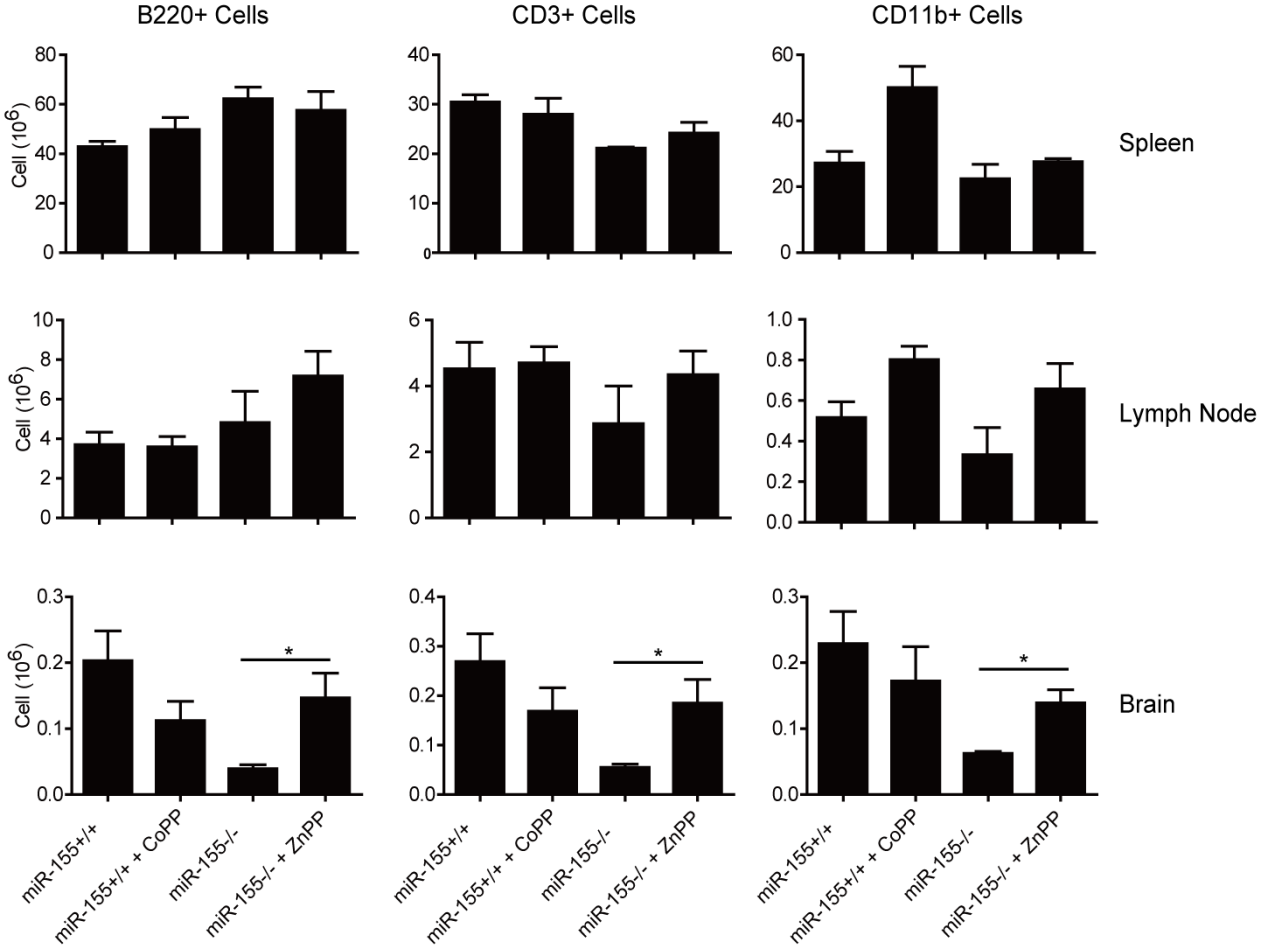
ZnPP treatment enhanced different cell types infiltrating into the brain of *miR-155* ^*-/-*^ mice. Cells were isolated from spleen, lymph nodes (LN) or brain 20 days after immunization. Numbers of B cells (B220^+^), T cells (CD3^+^) and monocytes/macrophages (CD11b^+^) were assessed by flow cytometry from spleen, LN and brain of *miR-155* ^*+/+*^ or *miR-155* ^*-/-*^ mice immunized with MOG_35-55_ and treated or not treated with CoPP or ZnPP. *: p<0.05. Data are shown as mean ±SEM. n = 6 mice per group. The results are representative of two independent experiments.

The pathogenesis of EAE initiates with immune cell infiltration into the brain [6]. Mice from the indicated groups were sacrificed and single cell suspensions were isolated from spleen, draining lymph nodes (LN) and brain on day 20 after immunization. The cellular content of each organ was characterized by flow cytometry. As depicted in Fig 2, the cellularity in brain was significant altered among the indicated four groups, whereas cellularity in spleen and lymph node were comparable. As expected, compared with *miR-155* ^*+/+*^ mice, the lack of miR-155 expression caused an important drop in the number of B220^+^ cells, CD3^+^ T cells and CD11b^+^ myeloid cells present within the brain. Inhibition of HO-1 by ZnPP, however, restored brain cellular infiltrates in *miR-155* ^*-/-*^ mice to numbers comparable to those observed in wild type counterparts, again supporting a role for HO-1 in regulating the inflammation of the central nervous system (CNS) in the absence of miR-155. However, induce HO-1 expression by CoPP treatment in *miR-155* ^*+/+*^ mice failed to modify the cellularity in the brain.

### HO-1 inhibition promotes Th1 and Th17 responses in CNS during EAE in *miR-155* ^*-/-*^ mice

**Fig 3:**
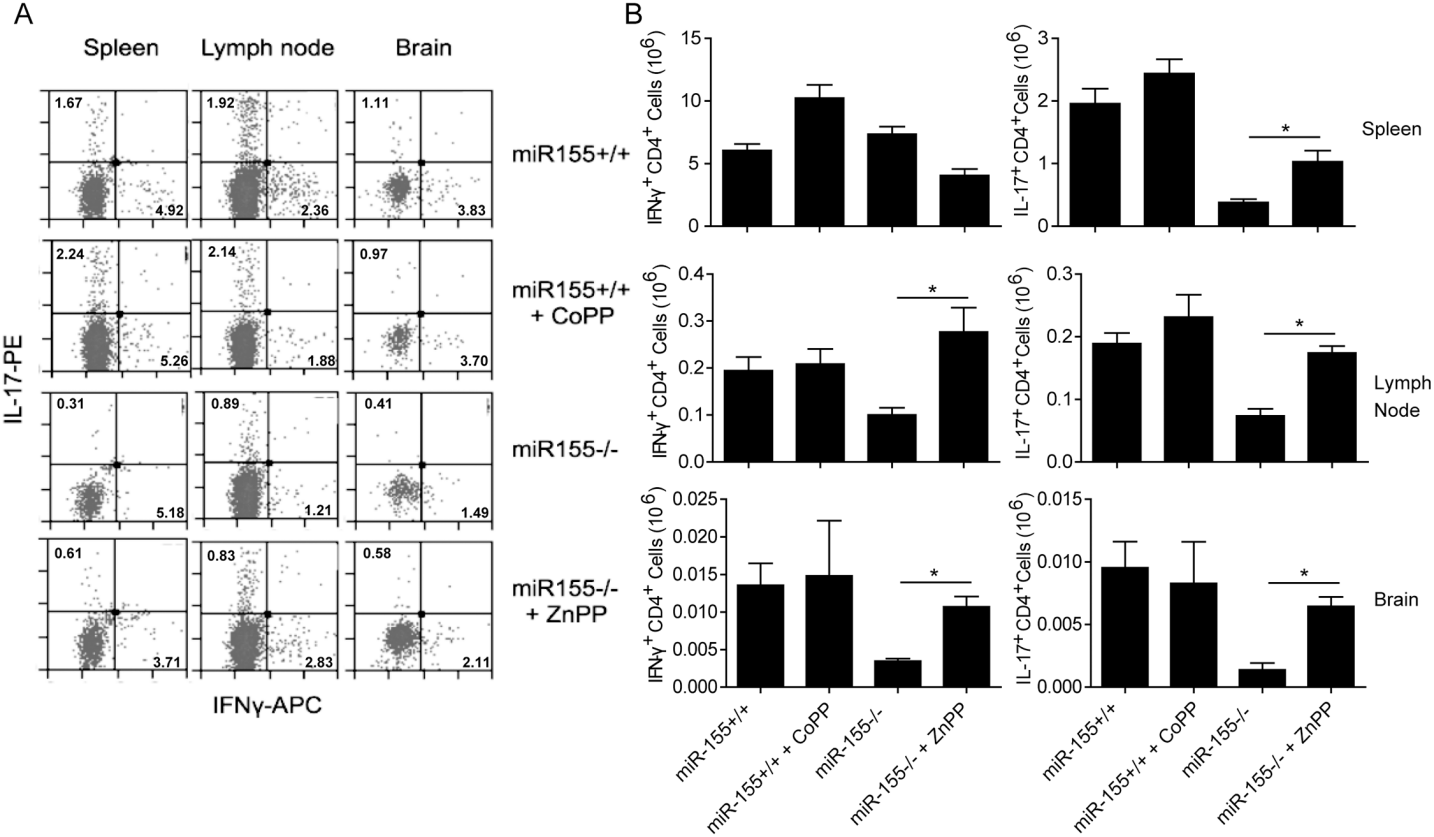
Th1 and Th17 responses in the spleen, LN or brain of *miR-155* ^*+/+*^ and *miR-155* ^*-/-*^ treat with CoPP or ZnPP after MOG_35-55_-immunization. (A) Flow cytometric analysis of IFN-γ and IL-17 expression in T cells (Gated on CD3 and CD4 double positive cells) from spleen, LN and brain of *miR-155* ^*+/+*^ or *miR-155* ^*-/-*^ mice immunized with MOG_35-55_ and treated or not treated with CoPP or ZnPP. Data are shown as mean ±SEM. n = 6 mice per group. The results are representative of two independent experiments. (B) Numbers of IFN-γ- or IL-17-producing T cell (Gated on CD3 and CD4 double positive cells) from spleen, LN and brain of *miR-155* ^*+/+*^ or *miR-155* ^*-/-*^mice immunized with MOG_35-55_ and treated or not treated with CoPP or ZnPP. *: p<0.05. Data are shown as mean ±SEM. n = 6 mice per group. The results are representative of two independent experiments.

During EAE, myelin peptide-specific Th1 and Th17 effector cells in CNS are responsible for disease pathology [15–16]. We then examined spleen, draining lymph node and brain for the presence of Th1 and Th17 cells during EAE. Results presented in Fig 3 show that both the numbers of Th1 and Th17 cells present in the lymph node or brain of *miR-155* ^*-/-*^ mice treated with ZnPP were very similar to those in vehicle-treated *miR-155* ^*+/+*^ mice. This contrasted sharply with the modest numbers of Th1 and Th17 cells present in spleen, lymph node or brain of *miR-155* ^*-/-*^ mice treated with vehicle only. On contrary, the total numbers of these inflammatory T cell populations in the LN and brain were similar in *miR-155* ^*+/+*^ treated with vehicle versus *miR-155* ^*+/+*^ mice treated with CoPP. Altogether, these observations reinforced the idea that miR-155 promotes T cell-mediated inflammation by targeting HO-1.

### T cell intrinsic role for miR-155 in the development of inflammatory T cells during EAE

**Fig 4:**
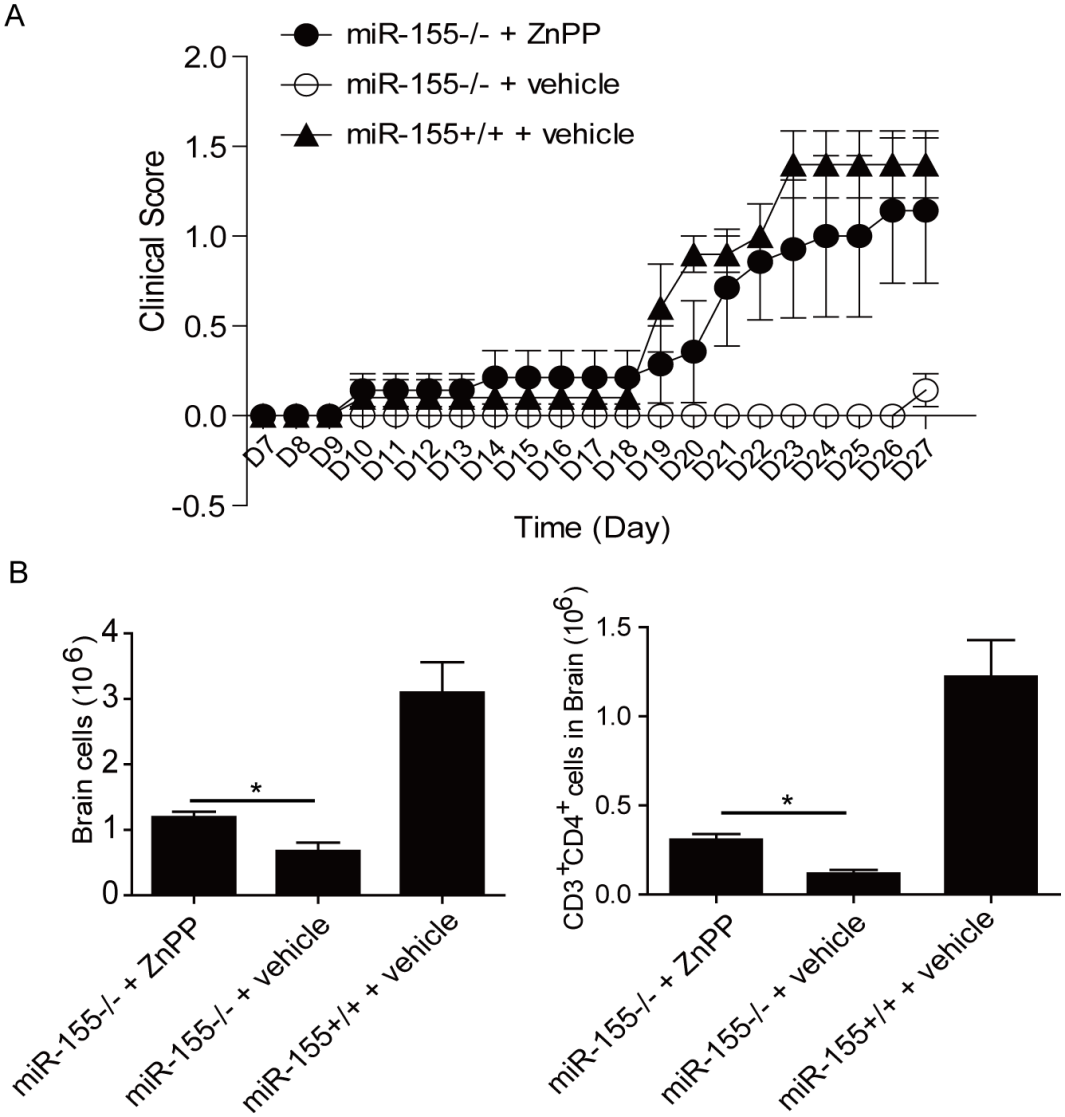
The failure of *miR-155* ^*-/-*^ CD4^+^ T cells to mediate EAE can be rescued by inhibiting HO-1. (A) *MiR-155* ^*-/-*^ CD4^+^ T cells or *miR-155* ^*+/+*^ CD4^+^T cells were transferred in *Rag1* ^*-/-*^ congenic recipients one day before EAE induction. Animals received daily ZnPP treatment or vehicle alone. Disease severity was scored based upon clinical symptoms. n = 7 mice per group. The results are representative of two independent experiments. (B) Total numbers of live cells (Left) and numbers of CD3^+^CD4^+^ cells (Right) in brain 27 days after immunization. *: p<0.05. Data are shown as mean ±SEM. n = 7 mice per group. The results are representative of two independent experiments.

To test whether the failure of *miR-155* ^*-/-*^ T cells to mediate EAE could be corrected by the inhibition of HO-1, we adoptively transferred 4x10^6^ purified naïve *miR-155* ^*-/-*^CD4^+^ T cells into *Rag1* ^*-/-*^ congenic recipients and induced EAE 24 hours later. One group of mice were treated with ZnPP, the second one received only the vehicle. *MiR-155* ^*+/+*^CD4^+^T cells transferred group was used as positive control. Mice receiving ZnPP had a substantially more severe and accelerated disease course compared to vehicle-treated mice (Fig 4A). At the peak of the diseases, mice from the three groups were sacrificed and cells from brain were separated. Consistently, increased numbers of cells were observed in the brain of mice that received ZnPP (Fig 4B).

**Fig 5:**
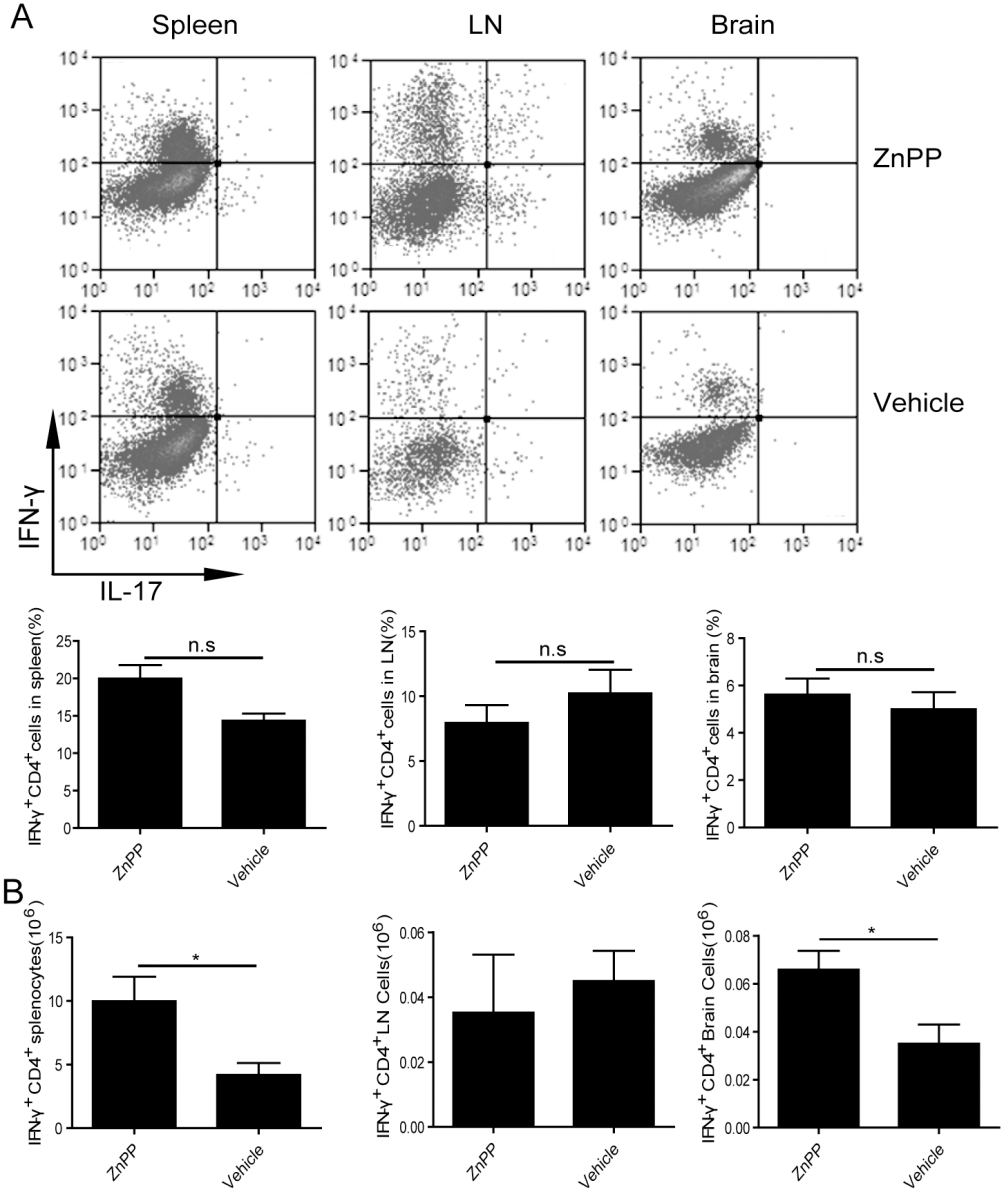
Increased Th1 cells infiltrating the brain of *miR-155* ^*-/-*^ mice treated with ZnPP. *MiR-155* ^*-/-*^ CD4^+^ T cells were transferred in *Rag1* ^*-/-*^ congenic recipients one day before EAE induction. Mice received ZnPP treatment or vehicle alone. (A) Flow cytometric analysis of IFN-γ and IL-17 expression in T cells (Gated on CD3 and CD4 double positive cells) from spleen, LN and brain of *miR-155* ^*-/-*^ mice immunized with MOG_35-55_ and treated with ZnPP or vehicle alone. n.s: p>0.05. Data are shown as mean ±SEM. n = 6 mice per group. The results are representative of two independent experiments. (B) Numbers of IFN-γ- or IL-17-producing T cell (Gated on CD3 and CD4 double positive cells) from spleen, LN and brain of *miR-155* ^*-/-*^ mice immunized with MOG_35-55_ and treated ZnPP or vehicle alone. *: p<0.05. Data are shown as mean ±SEM. n = 6 mice per group. The results are representative of two independent experiments.

We also examined spleen, draining lymph node and brain for the presence of Th1 and Th17 CD4^+^ T cells in the indicated groups. Results presented in Fig 5A showed that the percentage of Th1 cells in the spleen, draining lymph node or brain of *miR-155* ^*-/-*^mice treated with ZnPP were very similar to those in vehicle-treated mice. However, the absolute number of Th1 cells in the spleen and brain of the ZnPP treated mouse was significantly increased than the vehicle treated mouse (Fig 5B). Altogether, these observations reinforced the idea that miR-155 can promote T cell-mediated inflammation by targeting HO-1 expression in T cells.

## Discussion

Immunization with MOG_35-55_ induces a chronic form of EAE in C57BL/6 mice, mostly mediated by CD4^+^ T cells [17]. More recently, the regulatory role played by miR-155 on T cell-mediated inflammation was established in EAE model [6]. Moreover, induction of HO-1 expression was also shown to suppress T cell-mediated neuro-inflammation and improve EAE in C57BL/6 mice immunized with MOG_35-55_ [11]. Interestingly, the same study demonstrated that blocking HO-1 by ZnPP treatment in *miR-155* ^*+/+*^ mice had no effect on EAE induction. Since we have previously shown that HO-1 is a target of miR-155, this situation could be attributed to the absence of HO-1 expression in chronically-stimulated *miR-155*-sufficient T cells. We confirmed that in vivo induction of HO-1 by repeated injection of HO-1 activator, CoPP, attenuated clinical signs of EAE in normal *miR-155* ^*+/+*^ mice. ZnPP treatment worsened the clinical signs of EAE in *miR-155* ^*-/-*^ mice. This observation is consistent with a role for miR-155 in repressing HO-1-mediated regulation of T cells and in promoting the development of EAE. Inhibition of HO-1 by ZnPP restored brain cellular infiltrates in *miR-155* ^*-/-*^ mice, again supporting a role for HO-1 in regulating the inflammation of the central nerve system in the absence of miR-155.

Though protoporphyrin treatment could restore antigen-driven expansion and tissue migration of chronical-stimulated *miR-155* ^*-/-*^ T cells, it did not improve the capacity of T cells to produce effector cytokines, such as IFN-γ [8]. In the adoptive transfer model, the percentage of Th1 cell was similar regardless of ZnPP treatment. However, the absolute number of Th1 cells was increased in ZnPP-treated group and the increased severity of the disease in *miR-155* ^*-/-*^ animals treated with the HO-1 inhibitor was caused by an enhanced T cell infiltration into the brain. Thus, miR-155-dependent regulation of HO-1 function primarily would appear to control T cell proliferation and migration to sites of inflammation. Interestingly, it has been well demonstrated that mitochondrial reactive oxygen species (mROS) signaling is required in T cells for activation of nuclear factor of activated T cells (NFAT) and subsequent IL-2 production [18]. Since lack of HO-1 induces mitochondrial oxidative stress and ROS production [19], one could hypothesize that miR-155-mediated control of HO-1 expression in T cells would be essential for IL-2-dependent proliferation and expansion following activation by antigen. This hypothesis is reinforced by the observation that HO-1 inhibition in T cells stimulates IL-2 production [8, 20].

We observed that, though ZnPP treatment induced EAE in *miR-155* ^*-/-*^ treated mice, it did not restore the clinical disease to wild-type levels. It is well established that microRNA usually targets the expression of many genes and that their overall effect on biological systems results from the regulation of multiple gene expression. Since HO-1 has been identified as one of miR-155 targets, we can hypothesize that treatment by ZnPP in our experiment does not regulate the anti-inflammatory function of other miR-155 targets and this situation could explain why HO-1 inhibition alone does not restore EAE to wild-type levels in *miR-155* ^*-/-*^ mice. Other miR-155 targets that have been identified are *Socs1*, *Smad* and *Ifngr* [21–23]. They all have been characterized for their anti-inflammatory function. Whether inhibition of their activity in conjunction with that of HO-1 could restore EAE in *miR-155*-deficient animals remain to be explored.

In conclusion, our work has confirmed the importance of miR-155 in T cells for inhibiting HO-1 expression and promoting T cell-driven inflammation leading to autoimmune disease. It remains, however, to evaluate precisely the mechanism by which HO-1 deletion promotes T cell-mediated EAE in *miR-155* ^*-/-*^ mice. This could help to explore novel and effective therapies against auto-immune diseases.

## Supporting Information

S1 Fig Inhibition of HO-1 had no effect on *miR-155* ^*+/+*^ mice in EAE. EAE was induced in *miR-155* ^*+/+*^ mice that received ZnPP or vehicle alone. Disease severity was regularly scored based on clinical symptoms (n = 7–10). Data are shown as Mean ± SEM. Data are representative of two independent experiments.
(TIF)
