# Supplementary figures and images for: Protoporphyrin Treatment Modulates Susceptibility to Experimental Autoimmune Encephalomyelitis in miR-155-Deficient Mice
Source: PLoS One. 2015 Dec 15;10(12):e0145237. doi: 10.1371/journal.pone.0145237 (PMC4684403; doi:10.1371/journal.pone.0145237)

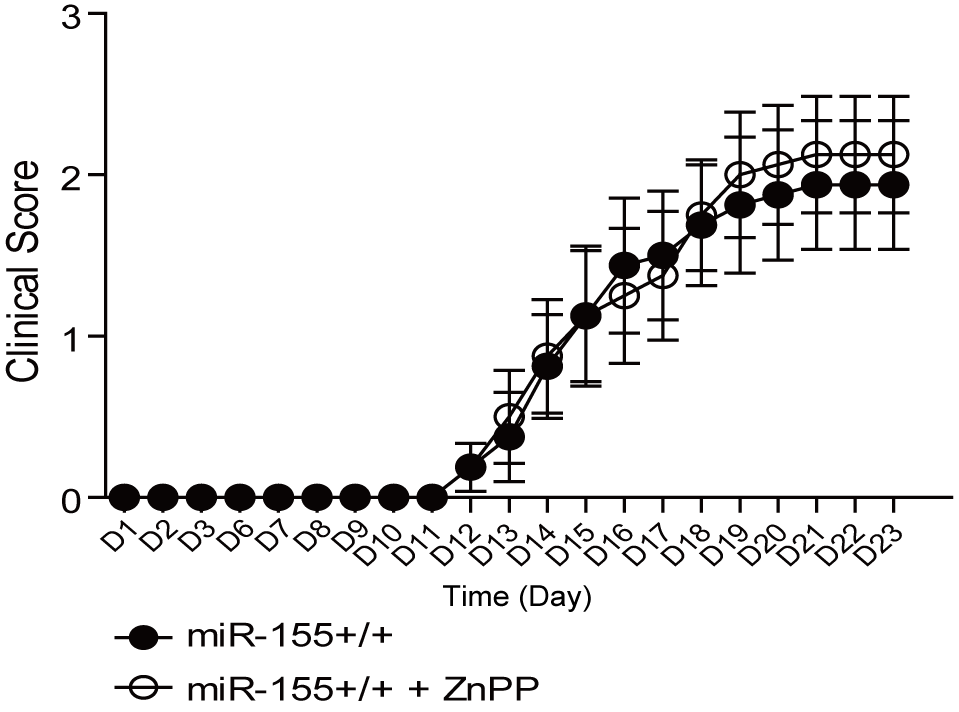

Supplement: S1 Fig — EAE was induced in miR-155 +/+ mice that received ZnPP or vehicle alone. Disease severity was regularly scored based on clinical symptoms (n = 7–10). Data are shown as Mean ± SEM. Data are representative of two independent experiments. (TIF) [file pone.0145237.s001.tif]
